# Laparoscopic ureterocalicostomy for ureteropelvic junction obstruction in a 10-year-old female patient: a case report

**DOI:** 10.1186/s13104-017-2569-x

**Published:** 2017-07-06

**Authors:** Yoko Nishimura, Kimihiko Moriya, Michiko Nakamura, Takeya Kitta, Yukiko Kanno, Hiroki Chiba, Masafumi Kon, Nobuo Shinohara

**Affiliations:** Department of Renal and Genitourinary Surgery, Hokkaido University Graduate School of Medicine, North-15, West-7, Kita-Ku, Sapporo, 060-8638 Japan

**Keywords:** Ureterocalicostomy, Laparoscopic surgery, Children

## Abstract

**Background:**

Ureterocalicostomy is indicated mainly in cases with failed pyeloplasty or with a completely intrarenal pelvis. While there have been several case series reported in adults, laparoscopic ureterocalicostomy in pediatric cases has rarely been reported. We report a case of pure laparoscopic ureterocalicostomy for ureteropelvic junction obstruction in an Asian female child.

**Case presentation:**

A 10-year-old female patient was referred to our hospital due to right high-grade hydronephrosis and a right renal stone, which was detected due to hematuria. Laparoscopic pyelolithotomy and ureterocalicostomy were indicated because of the completely intrarenal pelvis with thinning of the cortex, especially at the lower calyx. A transperitoneal approach was implemented in a lateral flank position with four trocars. After exposing the renal hilum, the renal stone was extracted without lithotripsy by making a small longitudinal incision at the ureteropelvic junction. Then, the ureter was transected, and the renal pelvis was closed. A 2-cm incision was made at the lower calyx. Uretero-caliceal anastomosis was completed in a running fashion using 5-0 absorbable sutures. The operation time was 379 min. The postoperative course was uneventful. Postoperative imaging studies showed marked improvement of the right hydronephrosis.

**Conclusion:**

Laparoscopic ureterocalicostomy is a safe and feasible treatment for selected patients with complicated ureteropelvic junction obstruction, even in the pediatric population.

## Background

Ureterocalicostomy (UC) has been reported as a surgical option for patients with complicated ureteropelvic junction obstruction (UPJO). Indications for this procedure include failed pyeloplasty or an intrarenal pelvis and other congenital anomalies of the kidney [1]. Open UC is a well-established procedure, and excellent outcomes have been reported [2–4]. While several case series of laparoscopic UC in adults have been published [5–7], laparoscopic UC in pediatric cases has rarely been reported. We report a case of pure laparoscopic UC for UPJO in a 10-year-old Asian female patient.

## Case presentation

**Fig. 1:**
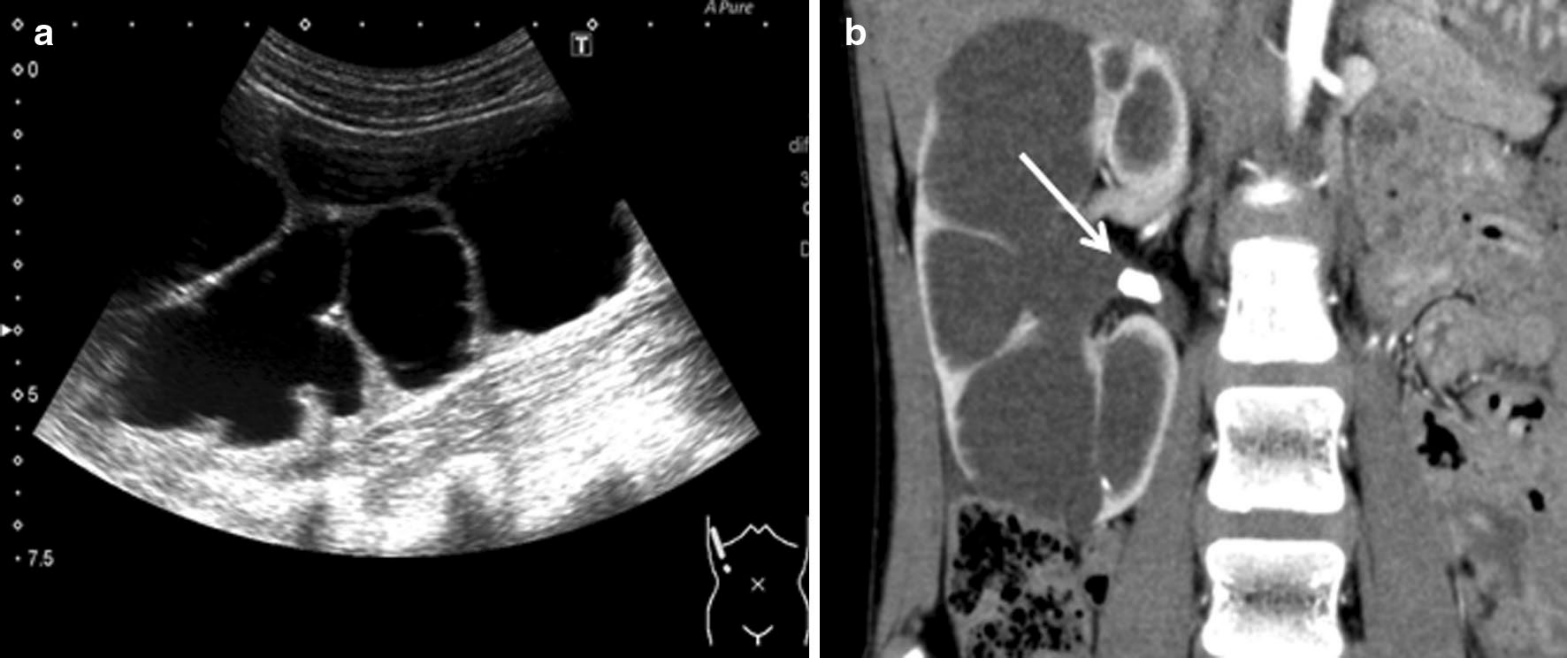
**a** Preoperative ultrasonography showed severe hydronephrosis. **b** CT scan revealed hydronephrosis and an 11 mm renal stone (*white arrow*) due to right ureteropelvic junction obstruction and thinning of the renal cortex

A 10-year-old female patient was referred to our hospital due to right high-grade hydronephrosis and a right renal stone, which was detected due to hematuria. Ultrasonography (US) and CT scan revealed hydronephrosis and an 11-mm renal stone (Fig. 1a, b). Technetium-99m mercaptoacetyltriglycine (MAG3) renography showed decreased relative function of the right kidney (right:left = 38.2:61.8). While a growth hormone was administered due to growth-hormone deficiency dwarfism, no metabolic abnormalities were detected as a cause of renal calculi in this patient.

Since urine stasis due to hydronephrosis was considered to be a potential cause of stone formation, we decided to perform surgical management for both the hydronephrosis and renal stone simultaneously. Laparoscopic UC and pyelolithotomy were indicated because of the completely intrarenal pelvis. Since the hydronephrosis was so severe with thinning of the cortex, especially at the lower calyx, bleeding due to incision at the lower pole could be controlled best under laparoscopy.

**Fig. 2:**
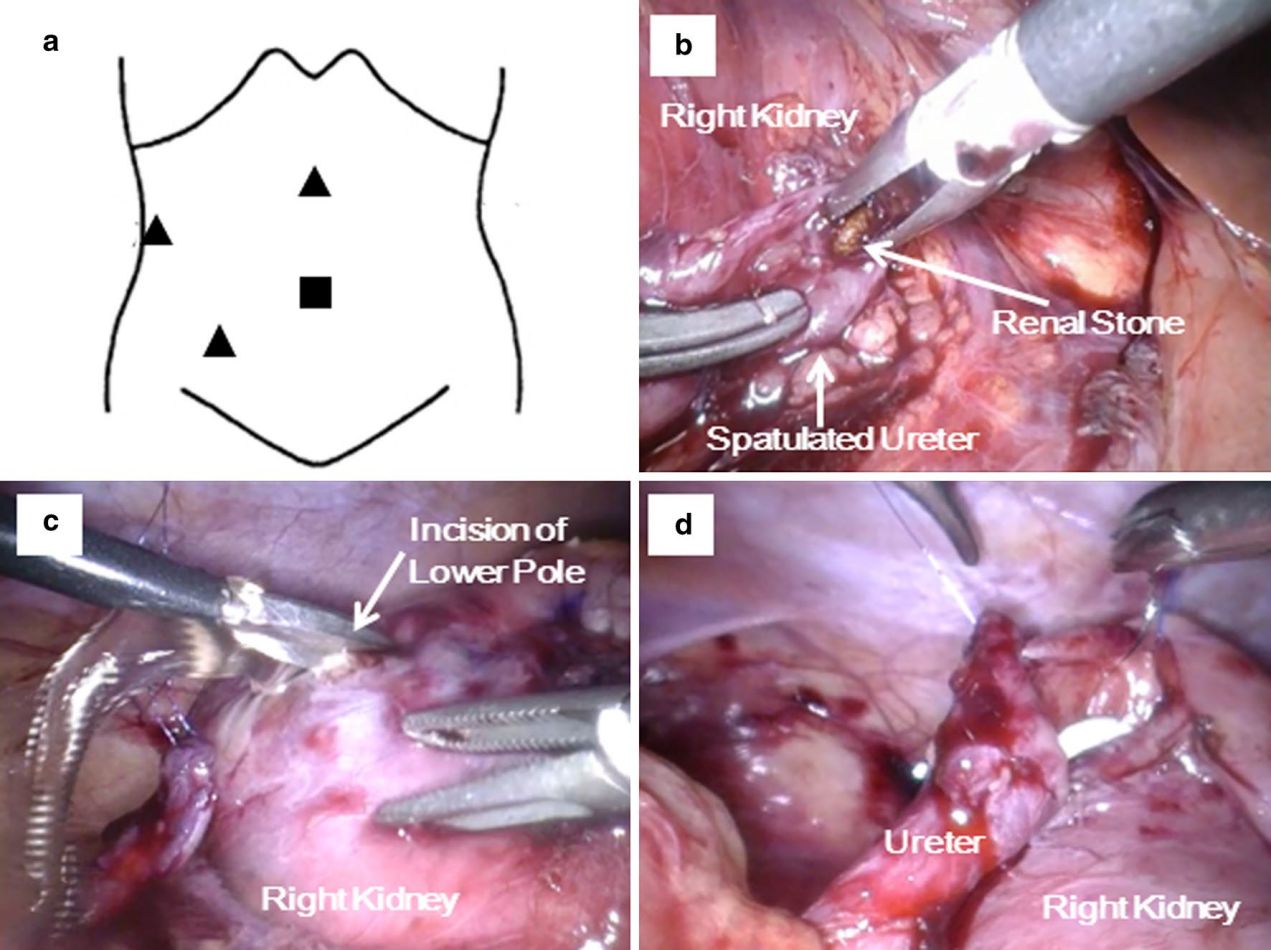
**a** A 12 mm camera port placed at the umbilicus (*square*) and three 5 mm working ports (*triangle*). **b** Pyelolithotomy without lithotripsy from the incision of the ureter. **c** Incision of the lower pole without renal pedicle clamping. **d** Uretero-caliceal anastomosis over the double-J ureteral stent

A transperitoneal approach was implemented in a lateral flank position under general anesthesia. Four trocars, including a 12-mm trocar at the umbilicus and three 5-mm trocars, were inserted in a similar fashion to laparoscopic pyeloplasty (Fig. 2a). After exposing the renal hilum and the upper ureter completely by reflecting the ascending colon, the renal stone was extracted without lithotripsy by making a small longitudinal incision at the ureteropelvic junction (Fig. 2b). A flexible ureteroscope inserted from the working port confirmed no residual stone in the right kidney. Then, the ureter was transected, and the renal pelvis was closed using 5-0 absorbable sutures at the level of the renal hilum.

To make the anastomosis, the thinning portion of the renal parenchyma at the lower calyx was identified using US, and a 2-cm incision was made with cold scissors (Fig. 2c). Bleeding from the incised thin renal parenchyma was minimal without clamping the renal vessels. Three anchor sutures were made using 5-0 absorbable sutures followed by insertion of a 5 Fr double-J ureteral stent in an anterograde manner. Uretero-caliceal anastomosis was completed in a running fashion using 5-0 absorbable sutures (Fig. 2d).

**Fig. 3:**
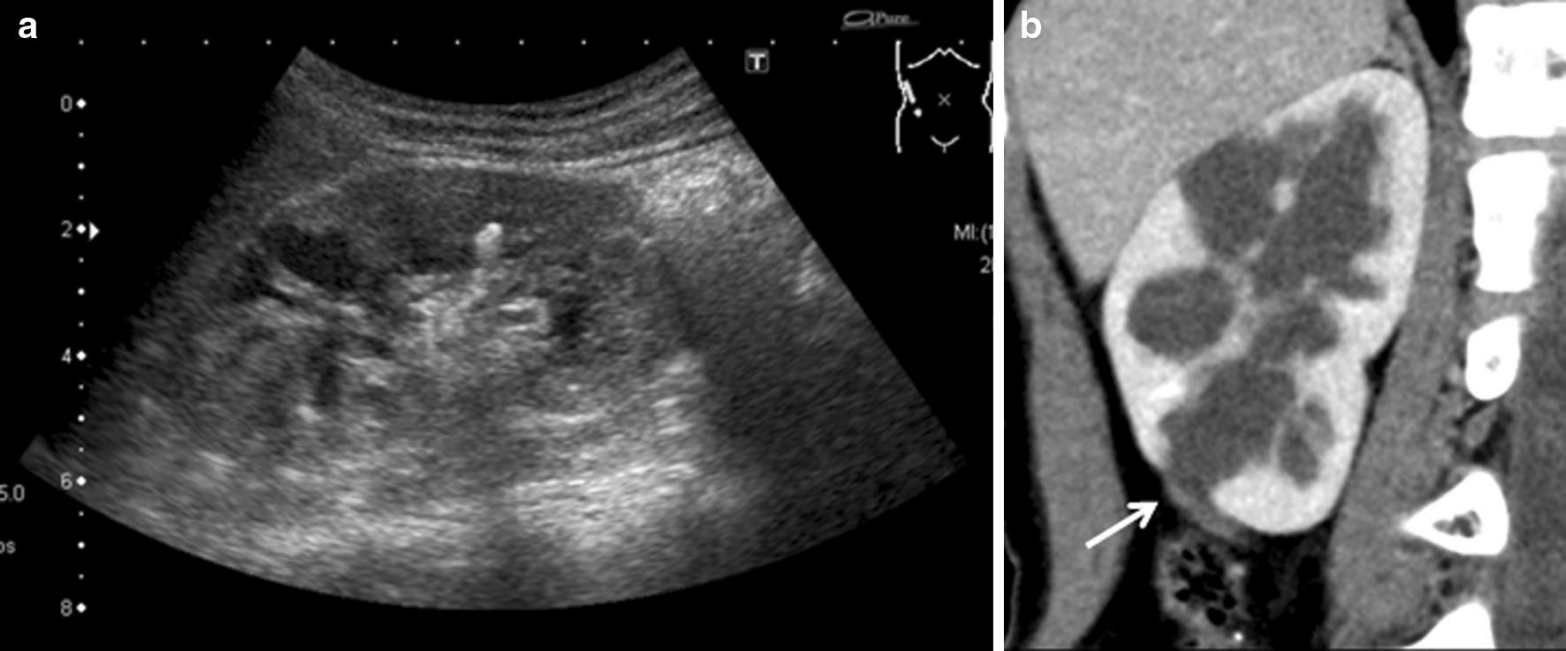
**a** Postoperative US showed improvement in the hydronephrosis 6 months after surgery. **b** CT scan revealed patency of the anastomosis (*white arrow*) at 15 months postoperatively

Blood loss was minimal, and no transfusion was required. The operation time was 379 min. The postoperative course was uneventful. An indwelling urethral catheter was removed 4 days after surgery, and the patient was discharged 6 days after surgery. Stone analysis revealed that the stone was composed of calcium oxalate and calcium phosphate. The double-J ureteral stent was removed 8 weeks postoperatively under general anesthesia. US performed at 6 months after surgery showed improvement of the right hydronephrosis (Fig. 3a). CT scan revealed patency of the anastomosis (Fig. 3b), and ipsilateral renal function as assessed by MAG3 renography at 15 months postoperatively had improved from 38.2% preoperatively to 42.0%. No reoccurrence of hydronephrosis or renal stones were observed for 29 months after the procedure.

## Discussion

Urolithiasis in children is often associated with underlying conditions such as metabolic abnormalities or genitourinary anomalies [8]. As no metabolic abnormalities were detected in our case, the possibility that a renal stone that was impacted at the ureteropelvic junction may have caused the renal dilatation rather than UPJO could not be ruled out. However, since urine stasis due to UPJO predisposes to the development of renal calculi [9, 10], we considered that pyeloplasty with pyelolithotomy, rather than percutaneous or transurethral lithotripsy alone, would be a more appropriate treatment in this case, in order to avoid the recurrence of renal calculi. Due to the completely intrarenal pelvis in the affected kidney, UC was consequently performed concurrently with laparoscopic pyelolithotomy in this 10-year-old female patient.

UC was initially reported by Neuwirt in 1948 [11]. Mesrobian et al. described that the indications for this procedure included previously unsuccessful pyeloplasty, UPJO associated with anomalies of renal rotation or ascent, an intrarenal pelvis or a short ureter [1]. The advantage of UC is that it provides dependent urinary drainage from the lower calyx into the ureter. However, bleeding from the incised renal parenchyma and the risk of anastomotic stricture are limitations of this procedure. Matlaga et al. reported 11 patients with successful open UC [2]. No patients experienced significant perioperative complications. Renal function in the affected kidney improved from a mean of 54.6% preoperatively to 60.1% postoperatively. Osman et al. reported that the success rate of 22 open UCs was 73% after a mean follow-up of 26.7 months [3]. They demonstrated that preoperative factors affecting the outcome of UC were a history of endopyelotomy or pyelonephritis, renal parenchymal thickening, split renal function and the extent of scarring score. Although only a few case series with limited numbers have been reported, open UC has shown favorable outcomes and was well-tolerated in the selected cases.

Laparoscopic UC in adults was first described by Gill et al. [5]. Subsequently, several case series about laparoscopic UC have been reported [5–7]. However, these were small series because the number of patients in whom UC is required is limited and because advanced laparoscopic skills, including the use of tension-free sutures and the ability to control bleeding of the incised renal parenchyma, are required for this procedure. Satisfactory outcomes were described in these reports as a low incidence of anastomotic stricture and improvements of drainage or renal function in the affected kidney after surgery.

Few reports regarding laparoscopic UC in the pediatric population have been published to date. Among 13 children described by Radford et al. an open approach and a laparoscopically assisted technique were indicated in 12 patients and in 1 patient, respectively [12]. To our knowledge, only 2 pediatric cases in a series by Arap et al. were treated via pure laparoscopic UC [7]. These patients were 2 and 8 years old and underwent the procedure after failed pyeloplasty. No intraoperative complications were observed. Each patient had a patent anastomosis and resolution of symptoms without significant worsening of split renal function.

In performing laparoscopic UC, control of bleeding from the anastomotic site is one of the most crucial issues [7]. In our case, the renal parenchyma at the lower calyx was thin enough to incise without hilar occlusion. Although there is no evidence of how thin the renal parenchyma should be for laparoscopic UC without hilar occlusion, this is a key factor in patient selection for laparoscopic UC. If laparoscopic UC is considered in cases with thick parenchyma at the anastomotic site, hilar occlusion as performed in partial nephrectomy [5] or open procedures should be indicated.

The other crucial issue for performing laparoscopic UC is a tension-free uretero-caliceal anastomosis. For this purpose, mobilization of the ureter while preserving vascular supply and a reliable suturing technique are essential. Recently, robotic-assisted laparoscopic UC has been reported [13, 14]. The advantages of robotics are three-dimensional visualization and increased freedom of movement compared to conventional laparoscopy. Accordingly, robotic-assisted laparoscopic UC may be a promising option even in the pediatric population, although it remains expensive.

## Conclusion

Laparoscopic UC is a safe and feasible treatment for selected patients with complicated UPJO, even in the pediatric population.

## Abbreviations

UC: ureterocutaneostomy
UPJO: ureteropelvic junction obstruction
US: ultrasonography
MAG3: Technetium-99m mercaptoacetyltriglycine
